# Correlation of lipocalin 2 and glycolipid metabolism and body composition in a large cohort of children with osteogenesis imperfecta

**DOI:** 10.1007/s40618-023-02121-4

**Published:** 2023-06-16

**Authors:** W.-b. Zheng, J. Hu, L. Sun, J.-y. Liu, Q. Zhang, O. Wang, Y. Jiang, W.-b. Xia, X.-p. Xing, M. Li

**Affiliations:** 1 Department of Endocrinology, Key Laboratory of Endocrinology, National Health and Family Planning Commission, Peking Union Medical College Hospital, Chinese Academy of Medical Sciences and Peking Union Medical College, Shuaifuyuan No. 1, Dongcheng District, Beijing, 100730 China; 2 Department of Endocrinology, Wuhan Union Hospital, Huazhong University of Science and Technology, Wuhan, 430022 Hubei China

**Keywords:** Lipocalin 2, Glycolipid metabolism, Muscle function, Body composition, Osteogenesis imperfecta

## Abstract

**Purpose:**

Lipocalin 2 (LCN2) is a newly recognized bone-derived factor that is important in regulation of energy metabolism. We investigated the correlation of serum LCN2 levels and glycolipid metabolism, and body composition in a large cohort of patients with osteogenesis imperfecta (OI).

**Methods:**

A total of 204 children with OI and 66 age- and gender-matched healthy children were included. Circulating levels of LCN2 and osteocalcin were measured by enzyme-linked immunosorbent assay. Serum levels of fasting blood glucose (FBG), triglyceride (TG), total cholesterol (TC), and low- and high-density lipoprotein cholesterol (LDL-C, HDL-C) were measured by automated chemical analyzers. The body composition was measured by dual-energy X-ray absorptiometry. Grip strength and timed-up-and-go (TUG) were tested to evaluate the muscle function.

**Results:**

Serum LCN2 levels were 37.65 ± 23.48 ng/ml in OI children, which was significantly lower than those in healthy control (69.18 ± 35.43 ng/ml, *P* < 0.001). Body mass index (BMI) and serum FBG level were significantly higher and HDL-C levels were lower in OI children than healthy control (all *P* < 0.01). Grip strength was significantly lower (*P* < 0.05), and the TUG was significantly longer in OI patients than healthy control (*P* < 0.05). Serum LCN2 level was negatively correlated to BMI, FBG, HOMA-IR, HOMA-β, total body, and trunk fat mass percentage, and positively correlated to total body and appendicular lean mass percentage (all *P* < 0.05).

**Conclusions:**

Insulin resistance, hyperglycemia, obesity, and muscle dysfunction are common in OI patients. As a novel osteogenic cytokine, LCN2 deficiency may be relevant to disorders of glucose and lipid metabolism, and dysfunction of muscle in OI patients.

**Supplementary Information:**

The online version contains supplementary material available at 10.1007/s40618-023-02121-4.

## Introduction

Lipocalin 2 (LCN2), also known as neutrophil gelatinase-associated lipocalin (NGAL), is a newly identified osteogenic cytokine, a secretory protein of a superfamily that bind and transport lipids and other hydrophobic molecules [1]. LCN2 is mainly produced by osteoblasts, which plays diverse functions, including anorexigenic action, reducing body weight, and improving the glucose metabolism through acting as a satiety signal molecule that is upregulated after feeding [1, 2]. LCN2 can cross the blood–brain barrier and activate the melanocortin four receptor (MC4R)-dependent pathway, one of the most potent regulating pathways of obesity [1]. However, the precise roles of LCN2 in diabetes, obesity, and muscle dysfunction still need to be elucidated in depth.

Over the years, metabolic dysfunction has been gradually noted in bone diseases. Osteogenesis imperfecta (OI) is the most common inherited disorders of connective tissue, which is characterized by increased bone fragility, impaired bone strength, recurrent bone fractures, and progressive bone deformities, with several extra-skeletal manifestations, such as blue sclera, dentinogenesis imperfecta, joint hypermobility, and hearing impairment [3, 4]. According to the clinical severity, OI was classified as five subtypes: mild OI (type I), perinatally lethal OI (type II), progressive deforming OI (type III), intermediate (type IV), and OI with hypertrophic callus (type V) [5]. Recently, multiple studies have found that insulin resistance, hyperglycemia, obesity, and sarcopenia are common comorbidities of OI patients [6–9], but the mechanism is unknown, and researches are scarce with regard to the effects of osteogenic cytokine and metabolic dysfunction in OI patients. It is worth studying whether the important osteogenic cytokine LCN2 participates in metabolic abnormalities of OI patients.

Therefore, we investigate serum levels of LCN2 and parameters of glycolipid metabolism, body composition, and muscle function in a large cohort of patients with OI, and analyze their relationship.

## Subjects and methods

### Subjects

This was a cross-sectional study conducted in endocrinology department of Peking Union Medical College Hospital (PUMCH) from May 2018 to April 2022. OI patients less than 18 years old and age- and gender-matched normal controls who came for health examination were enrolled. The patients were diagnosed as OI if they met one of the following inclusion criteria: (1) patients had a genetic diagnosis of OI; (2) patients had a history of more than one bone fracture under minor trauma, and an age- and sex-adjusted BMD *Z*-score less than or equal to − 1.0 at lumbar spine (LS) or femoral neck (FN); (3) patients with BMD *Z*-scores less than or equal to − 2.0 at LS or FN [10, 11]. Patients were excluded if they had a history of fracture within the recent 6 months prior to enrollment, to exclude the impact of recent fracture on parameters of osteogenic cytokine and metabolism. Patients were also excluded if they had other inherited or metabolic bone disease, or receiving treatment which could affect the glycolipid metabolism or muscle function, or with severe dysfunction of liver or kidney.

Medical history of OI patients was obtained in detail, and the height and weight were measured by Harpenden stadiometer (Seritex Inc., East Rutherford, NJ, USA). Body mass index (BMI) was calculated as weight (kg) divided by the square of height (m). Standard deviation score (SDS) of height, weight, and BMI were calculated basing on the reference data in normal Chinese [12–14]. Overweight and obesity were defined as BMI higher than the 85th and 95th percentile of reference data of normal Chinese children [12]. Ambulatory status was recorded and included unassisted, exclusively wheelchair, and assisted devices (walkers). If the patients were unassisted, they were recorded as “ambulatory”, otherwise they were recorded as “nonambulatory”.

The study was approved by the Scientific Ethics Committee of PUMCH (JS-2081). The legal guardians of OI patients and the normal controls provided written informed consents before they participated in this study.

### Measurement of the levels of LCN2 and metabolic parameter

Venous blood specimens were obtained from OI patients and healthy controls between 8:00 and 9:00 am after an overnight fast. As osteocalcin (OC) is a very important marker of osteoblastic activity, serum OC levels were also detected. Serum LCN2 and OC levels were measured by enzyme-linked immunosorbent assay (ELISA, R&D Systems, Inc, USA) following the manufacturer’s instructions. The detection range of LCN2 and OC was 0.156–10.0 ng/ml and 0.5–16.0 ng/ml, respectively. The intra-assay coefficients of variation (CV) of LCN2 and OC were 3.1–4.4% and 3.0–4.8%, with the inter-assay CVs of 5.6–7.9% and 0.7–2.4%, respectively.

Serum levels of fasting blood glucose (FBG), triglyceride (TG), total cholesterol (TC), low-density lipoprotein cholesterol (LDL-C), high-density lipoprotein cholesterol (HDL-C), calcium (Ca), phosphate (P), total alkaline phosphatase (ALP, a bone formation marker), alanine aminotransferase (ALT), and creatinine (Cr) were measured by automated chemical analyzers (ADVIA1800, Siemens, Germany). Serum levels of fasting insulin (FINS), beta cross-linked carboxy-terminal telopeptide of type I collagen (β-CTX, a bone resorption marker), 25-hydroxyvitamin D (25OHD), and intact parathyroid hormone (PTH) were measured using an automated electrochemiluminescence system (E170; Roche Diagnostics, Switzerland). Insulin sensitivity/resistance and β-cell function were calculated as follows: homeostasis model assessment insulin resistance (HOMA-IR) equal to FBG (mmol/L) × FINS(μU/mL)/22.5, and homeostasis model assessment islet beta cell function (HOMA-β) equal to 20 × FINS(μU/mL)/ (FPG, mmol/L)-3.5)) (%). As renal function could affect the results, Schwartz equation was used to estimate glomerular filtration rate (eGFR) [15].

Normal controls only measured serum levels of LCN2 and glycolipid metabolic parameters. All these biochemical parameters were measured in the clinical central laboratory of PUMCH.

### Measurement of BMD and body composition

BMD at lumbar spine (LS) 2–4 and proximal hip of OI patients were measured by dual-energy X-ray absorptiometry (DXA, Lunar Prodigy Advance, GE Healthcare, USA), of which Z-scores were calculated basing on a reference data of BMD in Chinese and Asian [13, 14]. Phantom testing was completed daily for calibration and quality check to ensure the accuracy of the measurement. The CVs of DXA measurement were 0.8 to 1.0%.

Composition of whole body of OI patients was also measured by DXA. Body fat mass percentage (%FM) and lean mass percentage (%LM) were calculated as the total fat mass or lean mass divided by the sum of bone, lean, and fat mass [16, 17]. Fat mass index (FMI) or lean mass index (LMI) was calculated as body fat mass (kg) or lean mass (kg) divided by height (m) squared, and appendicular mass was calculated as the sum of upper and lower limb masses [16, 17]. For males, slightly-to-moderately or severely increased %FM were set to greater than or equal to 20% or 25%, respectively, which were set to greater than or equal to 30% or 35% for female [18].

### Measurement of muscle function

Grip strength of the dominant hand was detected using a handheld dynamometer (Hand Grip Dynamometer, FEINECE. Inc, China), and the highest of three attempts was recorded. Physical function was measured by the timed-up-and-go (TUG) test. The patients were timed, while they rise from an arm chair with approximate height of 46 cm, walking at a comfortable and safe pace to a line on the floor 3 m away, turning and walking back to the chair and siting down again [19].

### Detection of pathogenic mutation of OI patients

Genetic mutations of OI patients were identified by a targeted next-generation sequencing (NGS) panel (Illumina HiSeq2000 platform, Illumina, Inc., San Diego, CA, USA) which was previously described in detail [20]. Twenty known candidate genes of OI were covered in this panel, including *COL1A1, COL1A2, IFITM5, WNT1, SERPINF1, FKBP10, CRTAP, SERPINH1, SP7, BMP1, PLS3, TMEM38B, PLOD2, P3H1, P4HB, PPIB, SEC24D, SPARC, CREB3L1,* and *MBTPS2*. The genomic DNA from OI patients were fragmented and ligated with end-repaired adaptors oligonucleotides. Then DNA fragments with adaptor molecules were purified and enriched by polymerase chain reaction (PCR). The candidate gene mutations identified by NGS were further confirmed by PCR and Sanger sequence.

### Statistical analysis

Continuous data of normal distribution (age, height, weight, BMI, eGFR, BMD, serum levels of LCN2, FBG, TC, TG, HDL-C, LDL-C, Ca, P, ALP, β-CTX, and Cr) were expressed as mean ± standard deviation (SD), while those of abnormal distribution (times of fracture, HOMA-IR, HOMA-β, serum levels of insulin, 25OHD, PTH, and ALT) were presented as median (quartiles). Categorical data were expressed as the number and percentage (%). The independent sample *T* test and the analysis of variance (ANOVA) were utilized to compare continuous data of normal distribution. Continuous data of abnormal distribution were analyzed by the Mann–Whitney *U *test and Kruskal–Wallis test for two groups or more groups. The Chi-squared test and Fisher’s test were used to analyze categorical variables. Relationships between the serum LCN2 levels and parameters of glycolipid metabolism were analyzed by the Spearman correlation. Multiple linear regression models were used for adjustments of age, gender, 25OHD, eGFR, ambulatory status, OC, and clinical classifications.

Statistical analyses were performed using the Statistical Package for Windows (version 20.0; SPSS Inc., Chicago, IL, USA). It was considered as significance if *P* value was less than 0.05.

## Results

### General characteristics and LCN2 levels of OI patients

A total of 204 OI children with mean age of 8.0 ± 4.6 years and 66 healthy children with age of 8.5 ± 3.6 years were included in this study. OI children had higher BMI and BMI SDS, more percentage of overweight and obesity, and higher serum FBG and OC levels, with lower HDL-C levels than control group (*P* < 0.05, Table 1).

**Table 1:** Metabolic disorder in OI children and healthy control

	OI patients	Healthy control	*P* value
	(*n* = 204)	(*n* = 66)	
Male, *n* (%)	145 (71.1%)	44 (66.7%)	0.497
Age (y)	8.0 ± 4.6	8.5 ± 3.6	0.461
Height (cm)	119.4 ± 28.1	133.0 ± 20.3	** < 0.001**
Height SDS	− 1.7 ± 2.6	0.5 ± 1.3	** < 0.001**
Weight (kg)	29.0 ± 16.2	32.4 ± 12.7	0.081
Weight SDS	− 0.2 ± 1.6	0.7 ± 1.1	** < 0.001**
BMI (kg/m^2^)	18.8 ± 3.9	17.6 ± 2.5	**0.004**
BMI SDS	1.15 ± 1.87	0.55 ± 1.63	**0.013**
Overweight, *n* (%)	10 (4.9%)	1 (1.5%)	**0.011**
Obese, *n* (%)	38 (18.6%)	4 (6.1%)	**0.014**
FBG (mmol/L)	5.07 ± 0.39	4.87 ± 0.42	**0.001**
Insulin (μIU/mL)	9.00 (6.00, 14.50)	7.35 (4.78, 12.95)	0.243
HOMA-IR	2.00 (1.34, 3.38)	1.53 (1.01, 2.80)	0.156
HOMA-β (%)	118.33 (80.00, 187.69)	117.50 (80.52, 170.63)	0.464
TC (mmol/L)	4.21 ± 0.79	4.28 ± 0.68	0.520
TG (mmol/L)	0.83 ± 0.45	0.78 ± 0.51	0.492
HDL-C (mmol/L)	1.04 ± 0.26	1.43 ± 0.29	** < 0.001**
LDL-C (mmol/L)	2.23 ± 0.57	2.25 ± 0.57	0.802
LCN2 (ng/ml)	37.65 ± 23.48	69.18 ± 35.43	** < 0.001**
OC (ng/ml)	24.15 ± 10.82	28.32 ± 11.58	**0.020**
ALT (IU/L)	14.0 (11.0, 18.0)	13.0 (11.0, 17.0)	0.608
Cr (μmol/L)	35.8 ± 12.3	47.8 ± 13.2	** < 0.001**
eGFR (ml/min/1.73 m^2^)	173.8 ± 41.2	150.1 ± 28.2	** < 0.001**

Bold values indicated that there was a significant difference between two groups

*OI* osteogenesis imperfecta, *BMI* body mass index, *SDS* standard deviation score, *FBG* fasting blood glucose, *HOMA-IR* homeostasis model assessment insulin resistance, *HOMA-β* homeostasis model assessment islet beta cell function, *TG* triglyceride, *TC* total cholesterol, *HDL-C* high-density lipoprotein cholesterol, *LDL-C* low-density lipoprotein cholesterol, *LCN2* lipocalin 2, *OC* osteocalcin, *ALT* alanine aminotransferase, *Cr* creatinine, *eGFR* estimated glomerular filtration rate

Serum LCN2 levels were 37.65 ± 23.48 ng/ml in OI children, which was significantly lower than those in control group (69.18 ± 35.43 ng/ml, *P* < 0.001). Serum LCN2 levels were similar between OI boys (36.47 ± 21.94 ng/ml) and OI girls (40.56 ± 26.89 ng/ml, *P* = 0.214).

### Association of LCN2 levels, metabolic abnormalities, and clinical severity of OI patients

**Fig. 1:**
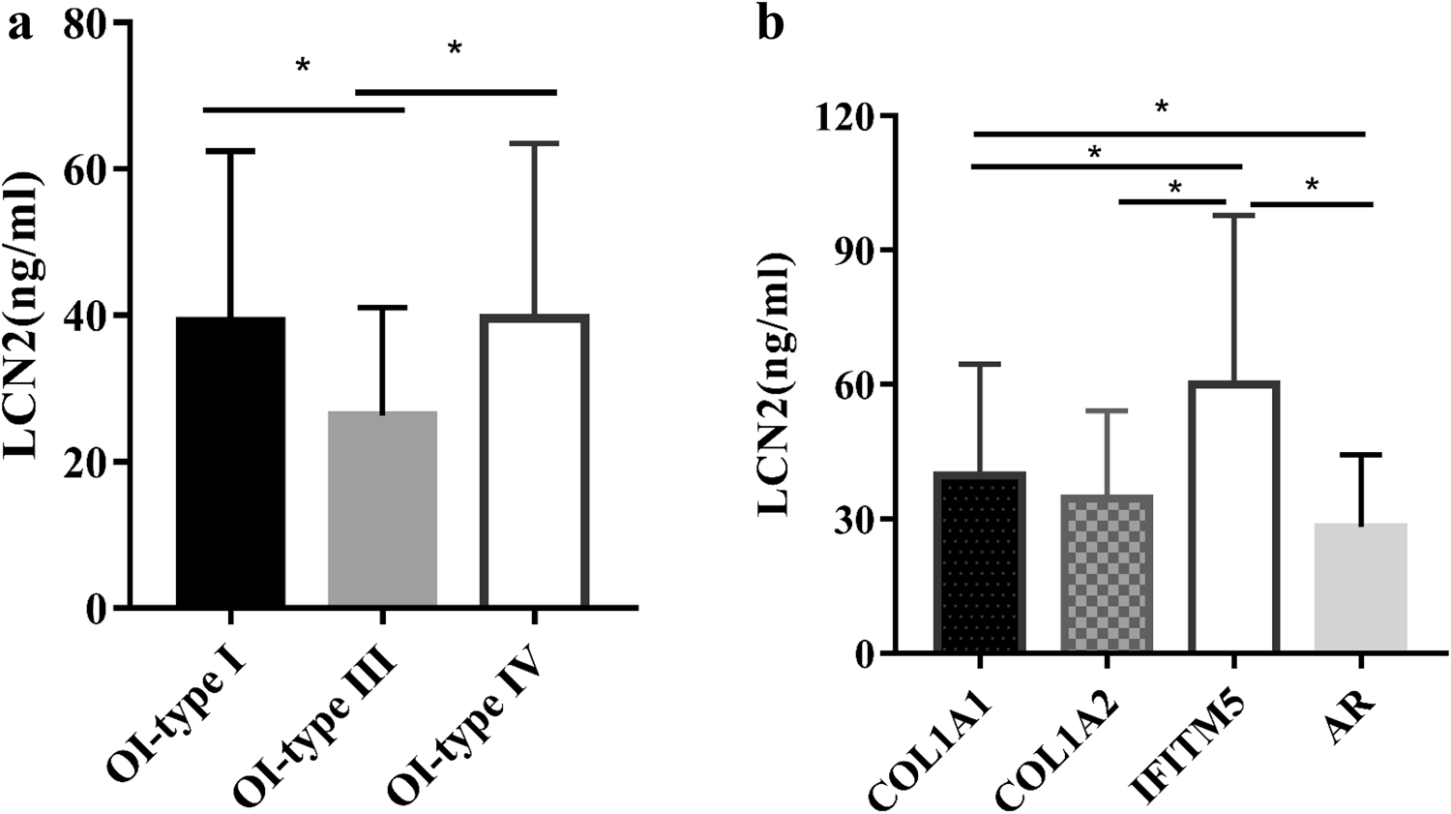
Serum LCN2 levels with different clinical and genotypic classifications of OI children. **a** Serum LCN2 levels with different clinical severity of OI children. **b** Serum LCN2 levels with different genotypes of OI children. ^*^P < 0.05

According to the clinical severity [21, 22], OI patients were classified as OI-type I, type III, type IV, and type V. OI-type III children had significantly lower LCN2 levels (26.23 ± 14.83 ng/mL) than children with type I (39.14 ± 23.29 ng/mL) and type IV OI (39.59 ± 23.89 ng/mL) (Fig. 1a, Table 2). Patients with OI-type III had higher BMI than type IV OI patients (*P* < 0.05), and significantly higher FBG levels than OI-type I and IV patients. HOMA-IR, HOMA-β, levels of TC, LDL-C, HDL-C, and TG had no differences among patients with OI-type I, III, and IV. These results indicated that the more severe of OI, the lower LCN2 levels, and the more obvious metabolic disorder.

**Table 2:** Metabolic disorder in OI children with different clinical severity

	Total OI patients	OI-type I	OI-type III	OI-type IV	*P* value
	(*n* = 204)	(*n* = 84)	(*n* = 36)	(*n* = 78)	
Male, *n* (%)	151 (67.1%)	64 (66.7%)^a^	21 (58.3%)^a,c^	62 (76.5%)^c^	**0.048**
Age (y)	8.0 ± 4.7	8.8 ± 4.5	7.4 ± 4.6	7.9 ± 4.6	0.249
Height (cm)	119.6 ± 27.9	128.3 ± 28.3^a,b^	101.9 ± 24.9^a,c^	119.5 ± 25.5^b,c^	** < 0.001**
Height *Z*-score	− 1.6 ± 2.6	− 0.9 ± 1.9^a^	− 4.0 ± 3.8^a,c^	− 1.6 ± 2.0^c^	** < 0.001**
Weight (kg)	29.0 ± 16.1	33.4 ± 17.2^a,b^	22.0 ± 12.2^a,c^	28.4 ± 15.7^b,c^	0.087
Weight *Z*-score	− 0.1 ± 1.5	0.3 ± 1.5^a,b^	− 1.4 ± 1.9^a,c^	− 0.3 ± 1.1^b,c^	** < 0.001**
BMI (kg/m^2^)	18.7 ± 4.0	18.8 ± 3.7	19.9 ± 4.8^c^	18.4 ± 3.6^c^	0.154
BMI SDS	1.15 ± 1.87	1.20 ± 1.80	1.23 ± 1.71	1.08 ± 1.86	0.880
Overweight, *n* (%)	10 (4.9%)	7 (8.3%)	2 (5.6%)	1 (1.2%)	0.121
Obese, *n* (%)	38 (18.6%)	12 (16.7%)	11 (30.6%)	14 (17.3%)	0.109
Nonambulatory, *n* (%)	78 (38.2%)	14 (14.3%)^a,b^	29 (80.6%)^a,c^	35 (43.2%)^b,c^	** < 0.001**
Number of fractures	4.0 (3.0, 7.0)	3.0 (2.0, 5.0)^a,b^	10.0 (4.3, 20.0)^a,c^	5.0 (3.8, 6.0)^b,c^	** < 0.001**
Ca (mmol/L)	2.46 ± 0.20	2.47 ± 0.09	2.48 ± 0.11	2.43 ± 0.30	0.427
P (mmol/L)	1.66 ± 0.20	1.69 ± 0.20	1.68 ± 0.21	1.64 ± 0.19	0.251
ALP (U/L)	285.7 ± 103.3	302.6 ± 106.2^a^	246.5 ± 84.6^a^	280.4 ± 93.9	**0.018**
β-CTX (ng/ml)	0.86 ± 0.36	0.91 ± 0.37^a^	0.67 ± 0.32^a^	0.85 ± 0.34	**0.044**
25OHD (ng/ml)	21.2 (13.7, 31.6)	21.4 (16.1, 31.5)	25.5 (17.2, 39.3)	22.3 (15.4, 27.8)	0.456
PTH (ng/ml)	21.2 (13.7, 31.6)	22.9 (17.7, 32.8)^a^	17.4 (10.5, 25.6)^a^	19.2 (11.1, 32.7)	**0.036**
ALT (U/L)	14.0 (11.0, 18.0)	14.0 (11.3, 19.0)^a^	12.0 (9.0, 16.0)^a,c^	14.0 (11.0, 19.0)^c^	0.054
Cr (μmol/L)	35.8 ± 12.3	38.7 ± 12.3^a,b^	33.1 ± 11.0^a^	34.6 ± 12.6^b^	**0.038**
eGFR (ml/min/1.73m^2^)	173.8 ± 41.2	174.2 ± 37.8^a^	157.6 ± 48.2^a,c^	190.7 ± 39.8^c^	**0.025**
FBG (mmol/L)	5.07 ± 0.39	5.00 ± 0.35^a^	5.27 ± 0.41^a,c^	5.04 ± 0.39^c^	**0.002**
Insulin (μIU/mL)	9.00 (6.00, 14.50)	7.90 (4.30, 13.80)	9.00 (7.40, 12.80)	10.50 (5.40, 17.90)	0.148
HOMA-IR	2.00 (1.34, 3.38)	1.79 (1.03, 3.16)	2.08 (1.68, 3.22)	2.36 (1.22, 4.03)	0.157
HOMA-β (%)	118.33 (80.00, 187.69)	109.23 (66.32, 181.25)	111.43 (92.50, 155.56)	142.35 (76.47, 250.00)	0.099
TC (mmol/L)	4.21 ± 0.79	4.14 ± 0.86	4.11 ± 0.65	4.31 ± 0.79	0.365
TG (mmol/L)	0.83 ± 0.45	0.82 ± 0.48	0.80 ± 0.39	0.85 ± 0.46	0.848
HDL-C (mmol/L)	1.04 ± 0.26	1.03 ± 0.26	1.07 ± 0.25	1.03 ± 0.26	0.795
LDL-C (mmol/L)	2.23 ± 0.57	2.19 ± 0.59	2.12 ± 0.41	2.33 ± 0.61	0.170
LCN2 (ng/ml)	37.65 ± 23.48	39.14 ± 23.29^a^	26.23 ± 14.83^a,c^	39.59 ± 23.89^c^	**0.007**
OC (ng/ml)	24.15 ± 10.82	25.93 ± 10.51^a^	17.84 ± 9.29^a,c^	24.95 ± 11.04^c^	**0.001**
LS BMD (g/cm^2^)	0.467 ± 0.199	0.547 ± 0.190^a,b^	0.341 ± 0.170^a,c^	0.448 ± 0.178^b,c^	** < 0.001**
LS BMD *Z*-score	− 2.2 ± 2.1	− 1.4 ± 1.8^a,b^	− 3.4 ± 2.1^a^	− 2.6 ± 1.9^b^	** < 0.001**
FN BMD (g/cm^2^)	0.427 ± 0.188	0.517 ± 0.190^a,b^	0.285 ± 0.160^a,c^	0.396 ± 0.146^b,c^	** < 0.001**
FN BMD *Z*-score	− 3.7 ± 2.3	− 2.7 ± 2.0^a,b^	− 5.3 ± 2.8^a,c^	− 4.2 ± 1.9^b,c^	** < 0.001**
Troch BMD (g/cm^2^)	0.349 ± 0.194	0.415 ± 0.204^a,b^	0.268 ± 0.187^a^	0.315 ± 0.163^b^	** < 0.001**
TH BMD (g/cm^2^)	0.471 ± 0.208	0.550 ± 0.223^a,b^	0.350 ± 0.186^a,c^	0.433 ± 0.163^b,c^	** < 0.001**

*OI* osteogenesis imperfecta, *BMI* body mass index, *SDS* standard deviation score, *Ca* calcium, *P* phosphate, *ALP* alkaline phosphatase, *β-CTX* *β cross-linked carboxy-terminal telopeptide of type I*, *25OHD* 25-hydroxyvitamin D, *PTH* parathyroid hormone, *FBG* fasting blood glucose, *HOMA-IR* homeostasis model assessment insulin resistance, *HOMA-β* homeostasis model assessment islet beta cell function, *TG* triglyceride, *TC* total cholesterol, *HDL-C* high-density lipoprotein cholesterol, *LDL-C* low-density lipoprotein cholesterol, *LCN2* lipocalin 2, *OC* osteocalcin, *ALT* alanine aminotransferase, *Cr* creatinine, *eGFR* estimated glomerular filtration rate, *LS* lumbar spine, *FN* femoral neck, *Troch* trochanter, *TH* total hip, *BMD* bone mineral density

Bold values indicated that there was a significant difference among three groups

^a^*P* < 0.05 for comparison between OI-type I and OI-type III; ^b^*P* < 0.05 for comparison between OI-type I and OI-type IV; ^c^*P* < 0.05 for comparison between OI-type III and OI-type IV

### Association of LCN2 levels and metabolic abnormalities with genotypes of OI patients

According to the inheritance pattern of pathogenic gene mutation, OI patients were divided into autosomal dominant (AD) inheritance*,* including *COL1A1*, *COL1A2*, *IFITM5* mutation, and the autosomal recessive (AR) inheritance, including mutations of *FKBP10*, *WNT1*, *TMEM38B*, *PLOD2*, *SERPINF1*, *BMP1*, *P3H1*, *SERPINH1*, and *CRTAP*. Since the patients with autosomal recessive inheritance were quite few, we summarize them into one group.

There was no significant difference in proportion of overweight or obesity, and serum levels of FBG, FINS, HOMA-IR, HOMA-β, TG, TC, LDL-C, and HDL-C among *COL1A1*, *COL1A2*, *IFITM5*, and the autosomal recessive (AR) gene mutation group (Table 3). However, children with *IFITM5* mutation had significantly higher serum LCN2 levels (60.1 ± 37.6 ng/mL) than the other gene mutation groups (*P* < 0.05). Children with *COL1A1* mutation had higher LCN2 levels (39.5 ± 25.1 ng/mL) than children with AR gene mutations (*P* < 0.05) (Fig. 1b, Table 3).

**Table 3:** Metabolic disorder in OI children with different genotypes

	*COL1A1*	*COL1A2*	*IFITM5*	AR	*P* value
	(*n* = 117)	(*n* = 60)	(*n* = 6)	(*n* = 21)	
Male, *n* (%)	83 (70.9%)	44 (73.3%)	3 (50.0%)	15 (71.4%)	0.694
Age (y)	8.0 ± 4.5^b^	8.4 ± 4.7^d^	3.7 ± 2.5^b,d,f^	8.2 ± 5.0^f^	0.123
Height (cm)	122.5 ± 27.5^b^	116.0 ± 28.4	97.2 ± 16.8^b^	117.8 ± 30.7	0.103
Height *Z*-score	− 1.2 ± 2.4^a^	− 2.6 ± 2.7^a^	− 0.6 ± 1.9	− 2.3 ± 3.0	**0.003**
Weight (kg)	30.1 ± 16.9^b^	27.9 ± 15.0	16.8 ± 8.5^b^	29.3 ± 16.6	0.236
Weight *Z*-score	0.0 ± 1.6^a^	− 0.6 ± 1.6^a^	− 0.5 ± 1.7	− 0.5 ± 1.3	**0.048**
BMI (kg/m^2^)	18.5 ± 3.8	19.2 ± 3.8	17.0 ± 5.3	19.7 ± 4.3	0.306
BMI SDS	0.99 ± 1.77	1.35 ± 1.88	0.86 ± 3.80	1.57 ± 1.68	0.428
Overweight, *n* (%)	8 (6.8%)	1 (1.7%)	0 (0)	1 (4.8%)	0.458
Obese, *n* (%)	20 (17.1%)	11 (18.3%)	1 (16.7%)	6 (28.6%)	0.666
Nonambulatory, *n* (%)	37 (31.6%)^a^	29 (48.3%)^a,e^	1 (16.7%)	11 (52.4%)^e^	0.155
Number of fractures	4.0 (3.0, 6.0)^a^	5.0 (3.0, 8.0)^a^	3.0 (1.8, 6.3)	4.0 (2.0, 8.5)	0.111
Ca (mmol/L)	2.45 ± 0.25	2.47 ± 0.12	2.48 ± 0.11	2.46 ± 0.14	0.897
P (mmol/L)	1.66 ± 0.19^b^	1.70 ± 0.23^d^	1.44 ± 0.15^b,d,f^	1.62 ± 0.15^f^	**0.011**
ALP (U/L)	304.3 ± 107.4^a^	244.8 ± 72.8^a,d^	343.8 ± 197.8^d^	281.8 ± 89.4	**0.002**
β-CTX (ng/ml)	0.87 ± 0.32^a,c^	0.71 ± 0.32^a,e^	0.89 ± 0.34	1.08 ± 0.52^c,e^	**0.006**
25OHD (ng/ml)	21.6 (15.3, 30.3)	22.8 (15.8, 27.1)	24.1 (15.1, 35.6)	25.4 (16.2, 34.9)	0.67
PTH (ng/ml)	21.4 (14.0, 31.0)	21.0 (12.1, 34.4)	16.9 (12.6, 34.1)	21.2 (14.2, 52.8)	0.931
ALT (U/L)	14.0 (11.0, 18.0)	14.0 (11.0, 18.5)	12.5 (9.5, 21.3)	14.0 (9.0, 23.0)	0.884
Cr (μmol/L)	36.0 ± 11.2	36.1 ± 14.1	27.0 ± 4.8	36.5 ± 13.7	0.364
eGFR (ml/min/1.73m^2^)	175.6 ± 40.2	171.8 ± 43.1	174.4 ± 44.3	168.7 ± 42.9	0.884
FBG (mmol/L)	5.02 ± 0.40	5.10 ± 0.37	5.23 ± 0.51	5.20 ± 0.38	0.165
Insulin (μIU/mL)	9.00 (5.35, 16.60)	9.45 (6.43, 13.05)	7.80 (4.53, 25.70)	9.75 (7.63, 15.65)	0.777
HOMA-IR	1.93 (1.17, 3.53)	2.12 (1.44, 3.23)	1.80 (1.04, 6.42)	2.25 (1.70, 3.61)	0.751
HOMA-β (%)	123.08 (75.51, 192.12)	113.62 (80.56, 181.54)	103.75 (70.70, 243.79)	130.00 (85.63, 212.91)	0.729
TC (mmol/L)	4.20 ± 0.75	4.24 ± 0.93	4.26 ± 0.67	4.17 ± 0.65	0.984
TG (mmol/L)	0.82 ± 0.44	0.88 ± 0.52	0.84 ± 0.38	0.73 ± 0.29	0.666
HDL-C (mmol/L)	1.06 ± 0.27	1.03 ± 0.23	1.14 ± 0.34	0.99 ± 0.27	0.652
LDL-C (mmol/L)	2.25 ± 0.55	2.21 ± 0.67	2.06 ± 0.34	2.21 ± 0.40	0.878
LCN2 (ng/ml)	39.7 ± 24.8^b,d^	34.6 ± 19.6^e^	60.1 ± 37.6^b,e,f^	28.3 ± 11.5^d,f^	**0.012**
OC (ng/ml)	24.73 ± 10.42	22.62 ± 8.76	26.04 ± 9.45	24.68 ± 17.47	0.634
LS BMD (g/cm^2^)	0.494 ± 0.194	0.441 ± 0.206	0.360 ± 0.157	0.425 ± 0.205	0.122
LS BMD *Z*-score	− 1.9 ± 1.9^c^	− 2.5 ± 2.0	− 2.8 ± 2.5	− 3.3 ± 2.5^c^	**0.022**
FN BMD (g/cm^2^)	0.457 ± 0.181^a^	0.374 ± 0.183^a^	0.438 ± 0.162	0.405 ± 0.220	**0.047**
FN BMD *Z*-score	− 3.3 ± 2.1^a^	− 4.5 ± 2.5^a^	− 2.7 ± 2.6	− 4.1 ± 2.6	**0.01**
Troch BMD (g/cm^2^)	0.363 ± 0.195	0.316 ± 0.175	0.304 ± 0.158	0.370 ± 0.245	0.437
TH BMD (g/cm^2^)	0.495 ± 0.206	0.435 ± 0.196	0.478 ± 0.167	0.438 ± 0.259	0.309

Bold values indicated that there was a significant difference among four groups

*AR* autosomal recessive, *BMI* body mass index, *SDS* standard deviation score, *Ca* calcium, *P* phosphate, *ALP* alkaline phosphatase, *β-CTX* *β cross-linked carboxy-terminal telopeptide of type I*, *25OHD* 25-hydroxyvitamin D, *PTH* parathyroid hormone, *FBG* fasting blood glucose, *HOMA-IR* homeostasis model assessment insulin resistance, *HOMA-β* homeostasis model assessment islet beta cell function, *TG* triglyceride, *TC* total cholesterol, *HDL-C* high-density lipoprotein cholesterol, *LDL-C* low-density lipoprotein cholesterol, *LCN2* lipocalin 2, *OC* osteocalcin, *ALT* alanine aminotransferase, *Cr* creatinine, *eGFR* estimated glomerular filtration rate, *LS* lumbar spine, *FN* femoral neck, *Troch* trochanter, *TH* total hip, *BMD* bone mineral density

^a^*P* < 0.05 for comparison between *COL1A1* and *COL1A2* mutation; ^b^*P* < 0.05 for comparison between *COL1A1* and *IFITM5* mutation; ^c^*P* < 0.05 for comparison between *COL1A1* mutation and AR group. ^d^*P* < 0.05 for comparison between *COL1A2* and *IFITM5* mutation. ^e^*P* < 0.05 for comparison between *COL1A2* mutation and AR group. ^f^*P* < 0.05 for comparison between *IFITM5* mutation and AR group

### Association of LCN2 levels and glycolipid metabolic parameters, body composition, and muscle function

**Fig. 2:**
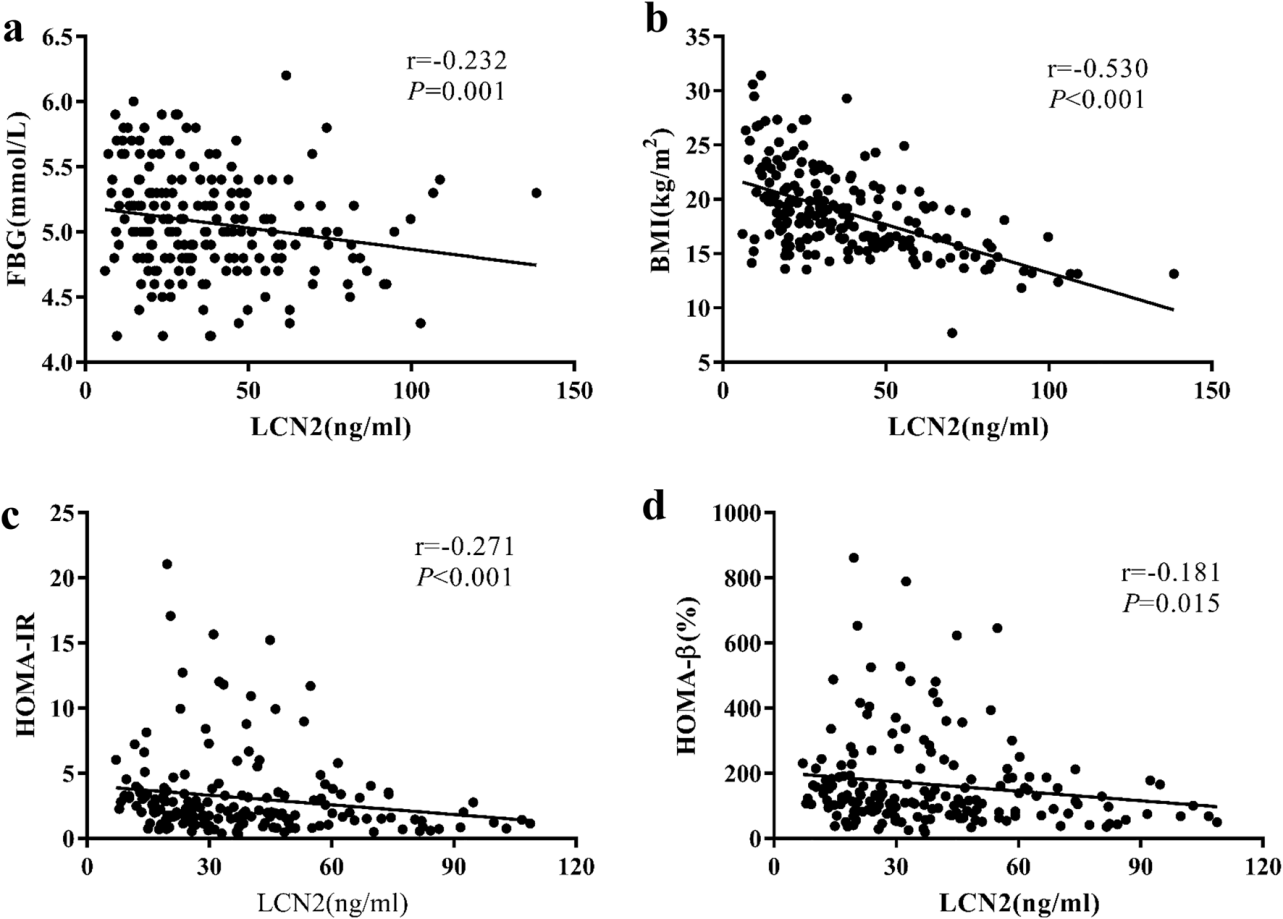
Correlation of serum LCN2 levels and glucose metabolic parameters in OI children. **a** Correlation of serum LCN2 levels and fasting blood glucose (FBG) in OI children. **b** Correlation of serum LCN2 levels and body mass index (BMI) in OI children. **c** Correlation of serum LCN2 levels and HOMA-IR in OI children. **d** Correlation of serum LCN2 levels and HOMA-β in OI children

Serum LCN2 levels were negatively correlated with age, BMI, and serum levels of FBG and insulin, HOMA-IR, and HOMA-β of OI children (Fig. 2, Table 4). Our previous study has found that osteocalcin had a close relationship with glycolipid metabolism in OI children [23]. Thus, when we analyzed the relationship between glucolipid metabolism parameters and LCN2, we adjusted age, gender, OC levels, 25OHD levels, eGFR, ambulatory status, and clinical classifications, and the negative correlation between levels of LCN2 with BMI, serum insulin levels, and HOMA-β remained (Supplementary Table 1).

**Table 4:** The correlation of LCN2 levels and metabolic parameters in OI children

	LCN2	
	*r*	*P* value
Gender	0.053	0.448
Age	− 0.202	**0.004**
BMI	− 0.530	** < 0.001**
FBG	− 0.232	**0.001**
Insulin	− 0.254	**0.001**
HOMA-IR	− 0.271	** < 0.001**
HOMA-β	− 0.181	**0.015**
TC	− 0.053	0.475
LDL-C	− 0.121	0.102
HDL-C	0.118	0.111
TG	− 0.067	0.368
Ambulatory status	− 0.265	** < 0.001**
Clinical classification	0.034	0.626
Times of fracture	− 0.059	0.398
ALP	0.197	**0.005**
β-CTX	0.171	**0.036**
OC	0.347	** < 0.001**
25OHD	0.048	0.584
PTH	0.051	0.533
LS BMD	− 0.029	0.677
FN BMD	0.018	0.798
Troch BMD	− 0.013	0.861
TH BMD	0.019	0.795
LS BMD *Z*-score	0.038	0.593
FN BMD *Z*-score	0.102	0.148
Cr	− 0.082	0.251
eGFR	0.038	0.594

*LCN2* lipocalin 2, *BMI* body mass index, *FBG* fasting blood glucose, *HOMA-IR* homeostasis model assessment insulin resistance, *HOMA-β* homeostasis model assessment islet beta cell function, *ALP* alkaline phosphatase, *β-CTX* β cross-linked carboxy-terminal telopeptide of type I, *25OHD* 25-hydroxyvitamin D, *PTH* parathyroid hormone, *FBG* fasting blood glucose, *HOMA-IR* homeostasis model assessment insulin resistance, *HOMA-β* homeostasis model assessment islet beta cell function, *TG* triglyceride, *TC* total cholesterol, *HDL-C* high-density lipoprotein cholesterol, *LDL-C* low-density lipoprotein cholesterol, *LCN2* lipocalin 2, *OC* osteocalcin, *ALT* alanine aminotransferase, *Cr* creatinine, *eGFR* estimated glomerular filtration rate, *LS* lumbar spine, *FN* femoral neck, *Troch* trochanter, *TH* total hip, *BMD* bone mineral density. Bold values indicated that the correlation was significantly different

**Fig. 3:**
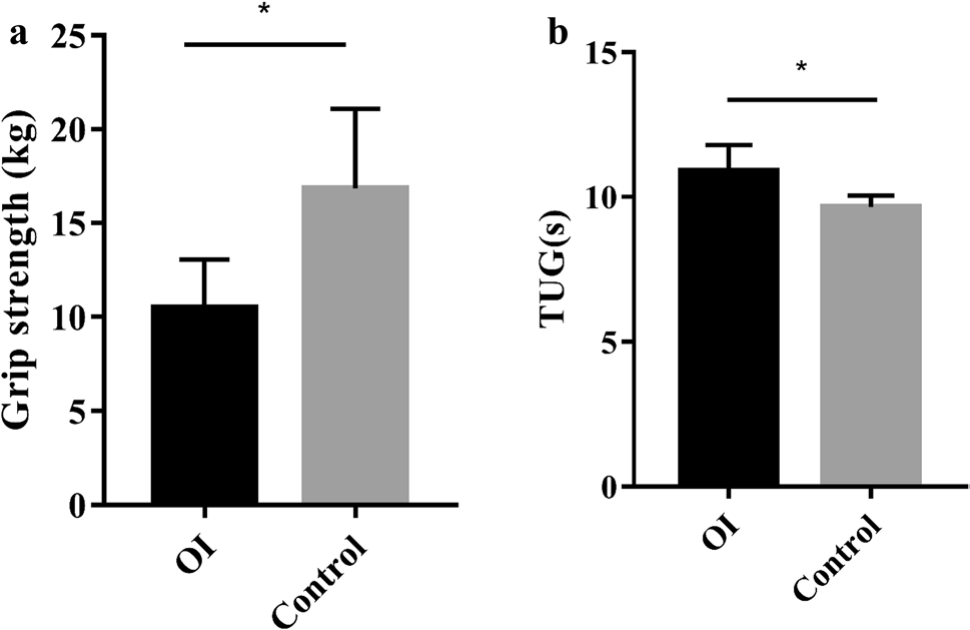
Muscle strength and function in OI children and healthy controls. **a** Grip strength in OI children and healthy controls. **b** Timed-up-and-go (TUG) test in OI children and healthy controls. ^*^P < 0.05

Body composition, the grip strength, and TUG test of 16 OI patients with age of 8.5 ± 3.6 years were measured. OI children had significantly lower grip strength and longer TUG than healthy children (Fig. 3, *P* < 0.05). We found that 1 (6.3%) and 10 (62.5%) patients had slightly-to-moderately or severely increased %FM, respectively. Serum LCN2 levels were positively correlated with total body and appendicular %LM, but negatively correlated to %FM and FMI of total body and trunk in OI children (Table 5). Serum LCN2 levels were uncorrelated to grip strength and TUG of OI children.

**Table 5:** The correlation between LCN2 levels with muscle parameters and body composition in OI children

	LCN2	
	*r*	*P* value
Grip strength	0.209	0.473
TUG	0.143	0.736
Total body LM (kg)	− 0.332	0.208
Total body LM%	0.662	**0.005**
Total body LMI	− 0.126	0.641
Appendicular LM (kg)	− 0.474	0.064
Appendicular LM (%)	0.656	**0.006**
Appendicular LMI	− 0.538	**0.031**
Total body FM (kg)	− 0.479	0.060
Total body FM%	− 0.656	**0.006**
Total body FMI	− 0.674	**0.004**
Trunk FM (kg)	− 0.471	0.066
Trunk FM%	− 0.653	**0.006**
Trunk FMI	− 0.594	**0.015**
Trunk/limb fat mass ratio	− 0.221	0.412

*TUG* timed-up-and-go, *LM* lean mass, *LMI* lean mass index, *FM* fat mass, *FMI* fat mass index. Bold values indicated that the correlation was significantly different

## Discussion

As LCN2 was recently identified as a critical bone-derived regulator of appetite, which was essential in regulating feeding behaviors and energy homeostasis, we examined serum LCN2 levels and analyzed its relationship with glycolipid metabolism and body composition for the first time in a relatively large cohort of OI children. We found that circulating LCN2 levels were significantly lower in OI children than in healthy children. We also found that the proportion of obesity and overweight, impaired glucose tolerance, and insulin resistance were significantly higher in OI children than in healthy children. More importantly, we observed for the first time that serum LCN2 levels were negatively correlated to BMI, FBG levels, HOMA-IR, HOMA-β, total body, and trunk fat mass percentage, and positively correlated to total body and appendicular lean mass percentage of OI children.

In this study, we found that the more severe the OI patient's clinical phenotype, the higher BMI they had, which was consistent with the previous findings [6, 7]. In a large North American cohort of OI children and adults, type III OI patients had higher BMI than type I and type IV OI patients [6]. Another study indicated that patients with type I and type III OI had higher FM% than normal controls [7]. These results suggested that the clinical severity of OI was closely correlated to a higher BMI and fat content of the body [7, 24]. In our study, we did not find that the pathogenic gene mutations were associated with OI children's body composition. However, we found that patients with *IFITM5* mutation had higher LCN2 levels than patients carrying other gene mutations, and the exact mechanism deserved further investigated.

As we know, obesity is an important risk factor for insulin resistance, diabetes, hypertension, hyperlipidemia, and atherosclerosis, and even higher mortality [25, 26]. We found that OI children had a higher proportion of obesity, overweight, and insulin resistance than normal children. BMI and serum FBG levels were also significantly higher and HDL-C levels were obviously lower in OI patients than in healthy controls. Previously, repeated fracture, bone deformation, and reduced activities were considered as the main causes of overweight and obesity in OI patients [24]. However, we found that the BMI was not correlated to the ambulatory status of OI patients in this study. This indicated that there could be other mechanisms involved in overweight, obesity, and metabolic disorders of OI patients.

On the other hand, in a relatively large sample study, lower muscle cross-sectional area was found in children and adolescents with OI-type I and type III than in healthy controls [27]. However, no correlation was found in the cross-sectional area of fat or muscle between OI patients with *COL1A1* and *COL1A2* mutations [27]. Likewise, we did not find that the pathogenic gene mutations were associated with OI children's body composition. Moreover, more and more studies indicated that the volume and strength of muscle were significantly reduced in OI patients [9, 28]. In this study, lower grip strength and longer TUG were found in OI children than in normal controls, which suggested that the muscle function of OI patients was weakened [8]. The mechanism of body composition changes and muscle dysfunction was also worth investigating in patients with OI.

In recent years, more and more evidence has shown that bone was also an endocrine organ, with osteoblasts and osteoblasts secreting bioactive factors with endocrine functions [29, 30]. It was previously thought that LCN2 was secreted mainly by adipose tissue, but recent studies have found that LCN2 was secreted by osteoblasts and its expression level in bone is at least tenfold higher than that in adipose tissue [1]. An important new function of bone was identified as regulation of food intake through osteogenic LCN2. Previous studies demonstrated that genetically or diet-induced obese animals had upregulated *Lcn2* gene expression in adipose tissue and liver [31, 32]. On the contrary, several studies showed that *Lcn2* knock out mice gained more weight, developed dyslipidemia and insulin resistance with high-fat diet compared to their wild-type littermates [33, 34]. In mice with osteoblast specific knockout *Lcn2* gene (*Lcn2 osb*^−/−^), serum levels of LCN2 were decreased by 67%, and impaired glucose tolerance, insulin resistance, and increased body weight, fat mass, and food intake was observed after glucose loading [1]. LCN2 was able to cross the blood brain barrier and binds to MC4R, thus played roles on the hypothalamus in primates, which could suppress food intake and reduce body weight [35, 36]. LCN2 was also described for playing roles in numerous processes, and increased hepatic gluconeogenesis and inflammatory state were found in LCN2-deficient mice [37]. In this study, we found that that serum LCN2 levels were significantly lower in OI children than in healthy children. We also found that serum LCN2 levels were negatively correlated to BMI, FBG levels, HOMA-IR, HOMA-β, total body, and trunk fat mass percentage, and positively correlated to total body and appendicular lean mass percentage of OI children. Therefore, we speculated that OI patients could had insufficient secretion of LCN2 from bone, which could increase food intake and then increase the risk of obesity and disorders of glucose metabolism. However, the mechanism of abnormal bone secretion of LCN2 in patients with OI is worthy of in-depth study.

Furthermore, the muscle strength and function were impaired in OI patients of this study, of which the disuse was previously considered as the main reason. A recent study indicated that serum LCN2 levels were increased following acute high‐intensity exercise [38]. The Wnt pathway antagonist, DKK1, and cytokine interleukin 6 were also increased after running, which not only played an important role in regulating muscle volume and muscle function, but also were positively correlated to LCN2 level. Besides, DKK1, RANKL, and TNF-α played key roles in regulating bone cell activity of subjects with OI [39]. *Lcn2*^−/−^ mice exhibited smaller muscle fibers, delayed muscle regeneration after injury, and impaired clearance of fibrous tissue from regenerated muscle, suggesting a novel role for LCN2 in regulating muscle satellite cell activation and skeletal muscle repair [38]. In this study, serum LCN2 level was positively correlated to total body and appendicular lean mass percentage of OI patients; therefore, we deduced that LCN2 may have a certain effect on muscle metabolism.

Thus, obesity, fasting hyperglycemia, insulin resistance, and decreased muscle strength were common comorbidities in OI children, which indicated that bones had important endocrine functions and there was a close crosstalk between bone, muscle, and adipose tissue. We speculated that OI led to reduced secretion of LCN2, which induce appetite increase and excess energy intake through regulating the activity of feeding center of hypothalamus, then leading to obesity, insulin resistance, hyperglycemia, hyperlipidemia, and muscle dysfunction. However, the present study had several limitations. This was a cross-sectional study, which made it difficult to establish a causal relationship between LCN2 levels and glycolipid metabolism and muscle function changes in OI patients. The information about the pubertal status and energy intake was not collected in OI children, which could play roles in glycolipid metabolism, body composition, peak bone mass, and peak muscle mass. Serum adipokines (such as asprosin and leptin) also played key roles in feeding behaviors and energy homeostasis and is important in the crosstalk between bone–muscle–pancreas–adipose tissues. We did not detect serum adipokines levels in OI patients, which was one of the limitations of this study. Moreover, body composition and muscle functions were measured only in a few OI patients of this study. Furthermore, we found that children with *IFITM5* mutation had significantly higher serum LCN2 levels than other gene mutation groups, and children with *COL1A1* mutation had higher LCN2 levels than children with autosomal recessive gene mutations, but the effects of different pathogenic gene mutations of OI on LCN2 secretion were still unknown.

In conclusion, obesity or overweight, insulin resistance, hyperglycemia, and sarcopenia are common comorbidity of OI children, of which circulating LCN2 deficiency may be closely relevant to these disorders, which indicates that bone may play an important role in regulating glycolipid metabolism and body composition through LCN2. Further studies on how LCN2 functions under different bone diseases are of great importance, and precise role of LCN2 in the pathogenesis of metabolic and muscular disorders will pave the way for novel therapies targeting LCN2.

### Supplementary Information

Below is the link to the electronic supplementary material.

Supplementary file1 (DOCX 16 KB)

## Data Availability

The data supporting the findings of the study are available from the corresponding author upon reasonable request.
