# Supplementary material for: Correlation of lipocalin 2 and glycolipid metabolism and body composition in a large cohort of children with osteogenesis imperfecta
Source: J Endocrinol Invest. 2023 Jun 16;47(1):47–58. doi: 10.1007/s40618-023-02121-4 (PMC10776749; doi:10.1007/s40618-023-02121-4)
Supplement: Supplementary file 1 — Supplementary file1 (DOCX 16 KB) [file 40618_2023_2121_MOESM1_ESM.docx]

**Supplementary Table 1. Multiple linear regression analysis to assess correlation between serum LCN2 levels with glycolipid metabolic parameters**

|  | BMI | |  | FBG | |  | Insulin | |  | HOMA-IR | |  | HOMA-β | |
| --- | --- | --- | --- | --- | --- | --- | --- | --- | --- | --- | --- | --- | --- | --- |
|  | β | *P* value |  | β | *P* value |  | β | *P* value |  | β | *P* value |  | β | *P* value |
| LCN2 | -0.427 | **<0.001** |  | -0.061 | 0.520 |  | -0.207 | **0.042** |  | -0.198 | 0.053 |  | -0.227 | **0.023** |

LCN2: lipocalin 2; BMI: body mass index; FBG: fasting blood glucose; HOMA-IR: homeostasis model assessment insulin resistance; HOMA-β: homeostasis model assessment islet beta cell function. Bold values indicated the correlation was significantly different.
